# Transcriptome sequencing for SNP discovery across *Cucumis melo*

**DOI:** 10.1186/1471-2164-13-280

**Published:** 2012-06-24

**Authors:** José Blanca, Cristina Esteras, Pello Ziarsolo, Daniel Pérez, Victoria FernÃ¡ndez-Pedrosa, Carmen Collado, Raquel RodrÃ­guez de Pablos, Alida Ballester, Cristina Roig, Joaquín Cañizares, Belén Picó

**Affiliations:** 1 Institute for the Conservation and Breeding of Agricultural Biodiversity (COMAV-UPV), Universitat Politècnica de València, Camino de Vera s/n, 46022, Valencia, Spain; 2 Sistemas Genómicos S.L, Ronda G. Marconi 6, 46980, Paterna, Valencia, Spain

## Abstract

**Background:**

Melon (*Cucumis melo* L.) is a highly diverse species that is cultivated worldwide. Recent advances in massively parallel sequencing have begun to allow the study of nucleotide diversity in this species. The Sanger method combined with medium-throughput 454 technology were used in a previous study to analyze the genetic diversity of germplasm representing 3 botanical varieties, yielding a collection of about 40,000 SNPs distributed in 14,000 unigenes. However, the usefulness of this resource is limited as the sequenced genotypes do not represent the whole diversity of the species, which is divided into two subspecies with many botanical varieties variable in plant, flowering, and fruit traits, as well as in stress response. As a first step to extensively document levels and patterns of nucleotide variability across the species, we used the high-throughput SOLiD™ system to resequence the transcriptomes of a set of 67 genotypes that had previously been selected from a core collection representing the extant variation of the entire species.

**Results:**

The deep transcriptome resequencing of all of the genotypes, grouped into 8 pools (wild African *agrestis*, Asian *agrestis* and *acidulus*, exotic Far Eastern *conomon*, Indian *momordica* and Asian *dudaim* and *flexuosus*, commercial *cantalupensis*, subsp. *melo* Asian and European landraces, Spanish *inodorus* landraces, and Piel de Sapo breeding lines) yielded about 300 M reads. Short reads were mapped to the recently generated draft genome assembly of the DHL line Piel de Sapo (*inodorus*) x Songwhan Charmi (*conomon*) and to a new version of melon transcriptome. Regions with at least 6X coverage were used in SNV calling, generating a melon collection with 303,883 variants. These SNVs were dispersed across the entire *C. melo* genome, and distributed in 15,064 annotated genes. The number and variability of *in silico* SNVs differed considerably between pools. Our finding of higher genomic diversity in wild and exotic *agrestis* melons from India and Africa as compared to commercial cultivars, cultigens and landraces from Eastern Europe, Western Asia and the Mediterranean basin is consistent with the evolutionary history proposed for the species. Group-specific SNVs that will be useful in introgression programs were also detected. In a sample of 143 selected putative SNPs, we verified 93% of the polymorphisms in a panel of 78 genotypes.

**Conclusions:**

This study provides the first comprehensive resequencing data for wild, exotic, and cultivated (landraces and commercial) melon transcriptomes, yielding the largest melon SNP collection available to date and representing a notable sample of the species diversity. This data provides a valuable resource for creating a catalog of allelic variants of melon genes and it will aid in future in-depth studies of population genetics, marker-assisted breeding, and gene identification aimed at developing improved varieties.

## Background

Melon (*Cucumis melo* L., Cucurbitaceae) is an important fruit crop worldwide. It is considered to be the most variable species in the genus *Cucumis*, and one of the most diverse among the cultivated vegetables [1,2]. Being most likely of African or Asian origin [3], melon is thought to have been first domesticated because of its nutritional seeds, with further selection having resulted in increased fruit and seed size. Melon has suffered an intense process of diversification and today exhibits a large variation in plant, flowering and fruit traits. Currently, the species comprises wild, feral and cultivated varieties, including sweet melons used for dessert and non-sweet ones consumed raw, pickled or cooked [4]. Wild melons are still frequent in East and West Africa, as well as from Central Asia to India. The main centers of diversity of melon are located between the Mediterranean basin (ranging from Southern and Eastern Europe to Turkey) and Central Asia (Iraq, Iran, Uzbekistan), and from India to the East Asian countries of China, Korea and Japan [5].

Traditionally, *C. melo* has been considered to be divided into two subspecies, *melo* and *agrestis*[6]. One of the simplest and most accepted classifications describes one single wild variety, var. *agrestis* Naud., and six cultivar groups (*cantalupensis* Naud., cantaloupe or muskmelon, *inodorus* Naud., cassaba and winter melons, *flexuosus* Naud., snake melons, *dudaim* Naud., mango melons, *momordica*, phoot or snap melons, and *conomon* Mak., pickling melon) [5,7]. More recently Pitrat et al. [8] split these varieties into 15 botanical groups (*cantalupensis, reticulatus, adana, chandalak, ameri, inodorus, chate, flexuosus, dudaim* and *tibish* (subsp. *melo*), *momordica, conomon, chinensis, makuwa,* and *acidulus* (subsp. *agrestis*)). However, some of these botanical groups are not well defined, share characteristics and are quite heterogeneous. Despite many reported accessions accurately fit into one of these distinctive taxonomic groups, other accessions displaying intermediated or mixed features are difficult to classify. *Cantalupensis* and *inodorus* are the botanical groups of greatest commercial interest. Both include different cultivar-types that are highly popular in different parts of the world.

Different marker systems have been used to assess the genetic diversity in melon by studying the genetic relationships among the different botanical groups (RFLPs, RAPDs, AFLPs, ISSRs and SSRs) (reviewed in Esteras et al. [2]). Most of the molecular studies strongly support the sub-specific division [9-11], reclassifying some of the botanical groups (the variety *tibish* has been included in the subspecies *agrestis*) and detecting a higher diversity among the *agrestis* types. In general, higher genetic diversity is reported in Africa and India than in the extremes of the distribution of melon (Mediterranean area and eastern Asia), which is consistent with the higher variation being maintained close to the center of domestication. The variability found in some groups of the subspecies *agrestis* (mostly *conomon* and *momordica*) has been used as a source of disease resistance for *cantalupensis* and *inodorus* cultivars and is also an underexploited reservoir of genetic variability for improving fruit quality in melon cultivars [4].

To date, the genetic basis of this diversity and the consequences of selection on genetic variation in the different wild and cultivated groups have not yet been studied on a genome-wide basis. The genomic abundance and amenability to cost-effective high throughput genotyping make single-nucleotide polymorphisms, SNPs, the most-used markers for genome-wide surveys of genetic diversity. Large SNPs collections have been identified in humans, several animals, and various model plants [12-19].

The availability of SNPs collections for melon has increased in the past few years with the sequences produced by several national and international projects using the Sanger technology [20-22]. Several thousand of SNPs were identified and some were mapped [10,23].

Second-generation sequencing (SGS) platforms, such as 454 GS FLX (Roche Applied Science), Solexa (Illumina Inc), and SOLiD (Life Technologies Inc), offer higher sequencing throughputs at greatly reduced costs. SGS platforms (mostly 454 and Solexa) are being used to resequence a number of genotypes in different crops (maize, rice, sorghum, soybean, common bean, brassicas, pumpkin, etc.), and are successfully generating vast amounts of SNPs. SGS is often combined with approaches to reduce genome complexity (genomic reduced representation libraries, transcriptome resequencing, etc.) [24,25]. SGS provides a reduced read length and lower per-base accuracy than data from Sanger sequencing. However, the 2-base encoding system used in the ligation-base sequencing protocol SOLiD ^TM^ enables a reduction of the sequencing error rate. This reduction translates into more accurate polymorphism discovery [26].

Blanca et al. [27] used SGS reads in melons for the first time to generate the latest and most complete version of the melon transcriptome, combining the previously available Sanger ESTs and the new sequences produced with the 454 platform (available at the NCBI Sequence Read Archive (SRA) with code SRA050214.1). A new and improved assembly of all these public ESTs (both Sanger and 454) is now available at the melogene database generated at the COMAV ( http://melogene.net).

In the study by Blanca et al. [27], the 454 platform allowed the deep transcriptome resequencing of a set of melon genotypes that were aligned to the reference transcriptome, yielding a large SNP collection in the species (a total of 38,587 SNPs). The genotypes included in this SGS-based SNP discovery assay represented the two most important melon market classes, the *inodorus* ‘Piel de Sapo’ and the *cantalupensis* “Charentais”, as well as the exotic *conomon* variety, which is mostly used for breeding. These markers are turning out to be extremely useful in the genetic diversity assays and breeding programs that use these varieties. This collection has been already used to construct a high-density genetic map employed to anchor and orient scaffolds in the melon whole genome sequence [28]. However, only 1 or 2 genotypes of each group were included, and therefore the within-group variability was not well represented. In addition, the other groups of the species were not represented in this first SGS sequencing assay.

To obtain a comprehensive overview of the sequence variation of melon genes, we have used SOLiD to resequence the transcriptome of 67 genotypes, grouped into 8 pools that represent all the botanical groups of the species. The completion of a draft of the genome sequence of melon [28] gives us the opportunity to mine SNVs on a genomic scale by using the reference genome for the alignment of short reads obtained by resequencing the variability across the species.

The diversity in African and Asian wild *agrestis* and exotic *acidulus* is analyzed here for the first time. Within the subsp. *melo*, we extended the study to better represent the variability of the *cantalupensis* group, the Spanish *inodorus* landraces, the Piel de Sapo commercial breeding lines, and also included the variability of melons from Eastern Europe and Western Asia that have not been represented in previous studies. Also, the intermediate group of *flexuosus*, *dudaim* and *momordica*, reservoir of resistance and quality genes for improving cultivated melons, has been analyzed. With this deep resequencing we captured a high number of SNVs between groups and detected some group-specific common variants. This new resource provides a unique opportunity to explore the genetic variation of melon and to identify sequence variants associated with phenotypes of interest.

## Methods

### Genotype selection

We used a core collection of 212 melon accessions, including wild relatives, feral types, landraces, breeding lines and commercial cultivars from 54 countries (representing the putative origin areas and diversity centers of the species). This collection was established on the framework of a previous project (MELRIP (2007–2010): ERA-PG project (GEN2006-27773-C2-2-E)), selfed, genotyped with AFLP markers and extensively phenotyped for plant and fruit traits at the COMAV [11]. Fifty two genotypes representing the variability of the species were selected on the basis of their molecular and phenotypic data. In this previous analysis we found a few discrepancies between the phenotype and the molecular results. Some accessions showing morphological features of a specific taxonomic group were molecularly similar to accessions of a different botanic group. Some others had intermediate features, reflecting the difficulties that sometimes arise during melon classification. In this paper, we employed for each accession the taxonomic group into which it was classified according to its phenotype, but the pooling strategy was decided combining phenotypic and previous AFLP results.

Additionally, 15 breeding lines belonging to 3 melon commercial market classes (two sets of *inodorus* lines, Piel de Sapo and Amarillo types, and one set of *cantalupensis* lines) were provided by Semillas Fitó (Barcelona, Spain) and included in the analysis. A total of 67 genotypes were resequenced. Some of these accessions have been used extensively as parental lines in breeding programs. The name, origin, and some phenotypic traits of the resequenced accessions are presented in Table 1, and photographs of each selected genotype are included in Additional file 1: “Resequenced melon genotypes”.

**Table 1:** Origin and characteristics of genotypes included in the 8 pools sequenced with SOLiD

**Genotype/collection code**	**Origin**	**Collection**	**Flower and fruit traits**	**% Mapped reads**	**Number of processed reads a**^**1**^
Subsp. *agrestis*					
Pool 1: African *agrestis*					
Tibish/CO199	Sudan	MELRIP	Mostly monoecious. Mostly small inedible fruits (<5 cm). Round to oval. Light green-white flesh. Non climacteric. No aroma. No sugar.	43.7	30,620,160
Fadasi/CO133	Sudan	MELRIP			
HSD/CO145	Sudan	MELRIP			
Tayer/CO195	Cameroon	MELRIP			
Agrestis/CUM 287	Nigeria	IPK			
Pool 2: Asian *agrestis- acidulus*					
Agrestis Wild chibbar/CO204	India	COMAV	Monoecious. Small to medium sized fruits. Oval, elliptic to elongated. Mostly non climacteric. White-light orange flesh. No aroma. No sugar. Low pH.	56.7	15,779,803
Acidulus SLK/CO187	Sri Lanka	MELRIP			
Agrestis Meloncito/CO153	India	COMAV			
Acidulus TGR 1551/PI 482420	Zimbabwe	NPGS			
Voatango/CO202	Madagascar	MELRIP			
Arya/CO115	India	COMAV			
Pool 3: Far East *conomon*					
**Pat81/CO32**	Korea	COMAV	Andromonoecious-hermaphroditic. Medium sized fruits. Flat, round to elongated. White-green-light orange flesh. Non climacteric-medium climacteric. Medium aroma. Medium sugar.	56.1	17,962,640
FreemansÂ´s Cucumber/CO136	Japan	COMAV			
**Songwhan Charmi/PI 161375**	Korea	NPGS			
Nabunkin/CO153	China	MELRIP			
Paul/CO169	Poland	MELRIP			
Intermediate types between subspecies					
Pool 4: Middle East and Indian *momordica*, *dudaim* and *flexuosus*					
Momordica/PI124112	India	NPGS	Andromonoecious-monoecious. Round, flat, oval to very elongated fruits. White-light orange flesh. Climacteric. No to intermediate sugar. Medium to strong aroma. Low pH.	55.3	23,320,668
Momordica/PI124111	India	NPGS			
Momordica/CUM 438	India	IPK			
Snakemelon/CO188	Saudi Arabia	MELRIP			
Flexuosus/CUM 353	Iraq	IPK			
Flexuosus/CUM 225	India	IPK			
Dudaim QueenÂ´s pocket melon/CO180	Afghanistan	COMAV			
Subsp. *melo*					
Pool 5: Group *cantalupensis*					
**Noy Israel/CO162**	Israel	COMAV	Andromonoecious-monoecious- gynoecious. Medium to large size fruits. Flat to oval. Round or ribbed. Green- orange flesh. Climacteric. Sweet. Aromatic.	48.1	23,237,004
Noir des carmes/CO161	France	COMAV			
Prescott Fond Blanc/CO179	France	COMAV			
TopMARK/NSL30032	USA	NPGS			
Nantais Oblong/CO159	France	MELRIP			
Gynadou/CO141	France	MELRIP			
CantalupdÂ´alger/CO121	France	MELRIP			
**PMR45/CO178**	USA	MELRIP			
5 Charentais breeding lines	Spain	S.Fitó S.A			
Pool 6: Group *melo* Eastern Europe, Central Asia*, inodorus, chandalack, ameri*					
Honeydew/CO143	USA	COMAV	Monoecious-andromonoecious. Medium-size fruits. Oval, flat to elongated. White-green-light orange flesh. Climacteric. Sweet, variable sugar content. Medium to low aroma.	34.3	8,367,385
Kirkagac/CO150	Turkey	COMAV			
Muchanesvi/CO156	Georgia	MELRIP			
Baskavas/CO118	Greece	MELRIP			
Korca/Cum168	Rusia	IPK			
Kiziluruk/CO96	Uzbequistan	COMAV			
Hami melon/CO142	China	COMAV			
Winter type/PI169329	Turkey	NPGS			
Maazoon/CO85	Egypt	COMAV			
Blanco/CO67	Spain	COMAV			
Carosello/CO122	Italy	COMAV			
Pool 7: *inodorus* Spanish landraces					
Cañadulce/CO48	Spain	COMAV	Andromonoecious. Large-sized fruits. Round to elliptic. White-Green flesh. Non climacteric. Sweet. Low aroma.	50.6	17,485,023
Madura amarilla/CO58	Spain	COMAV			
Erizo/CO75	Spain	COMAV			
Amarillo oro/CO79	Spain	COMAV			
Escrito oloroso/CO50	Spain	COMAV			
Tendral/CO59	Spain	COMAV			
Verde pinto/CO73	Spain	COMAV			
Coca/CO49	Spain	COMAV			
Mochuelo/CO48	Spain	COMAV			
Largo de Villaconejos/CO69	Spain	COMAV			
5 Amarillo breeding lines	Spain	S.Fitó S.A			
Pool 8: *inodorus* group market class Piel de sapo					
**T111**	Spain	S.Fitó S.A	Andromonoecious. Large-sized fruits. Round to elliptic. White-green flesh. Non climacteric. Sweet. No aroma.	37.2	13,809,773
5 Piel de Sapo breeding lines	Spain	S.Fitó S.A			

a^1^ Total number of reads and percentage of reads mapped on to the reference melon genome for SNP mining.

Genotypes marked in bold letters are those used for SNP mining trough transcriptome resequencing with Sanger and 454 in previous assays [20,21,27]. Seed source codes: IPK: Institut ftir Pflanzengenetik und Kulturpflanzenforschung, Gatersleben, Germany; NPGS: GRIN NPGS, National Plant System, USDA, USA; MELRIP (2007–2010): ERA-PG project (GEN2006-27773-C2-2-E); COMAV, Institute for the Conservation and Breeding of Agricultural Biodiversity, UPV, Spain; Semillas Fitó S.A. (Barcelona).

We prepared 8 pooled RNA samples. Three pools represented the variability of the subsp. *agrestis* (Table 1): the first RNA sample was prepared from 5 African genotypes, most belonging to the variety *agrestis* which is characterized by its small, inedible, non-climacteric fruits (<5 cm) (Additional file 1), with no sugar and no aroma, as well as another genotype belonging to the newly reported African variety *tibish*[8]; the second sample consisted of RNA from 6 genotypes, mostly Asian, of the *agrestis* and *acidulus* varieties, with traits similar to the first pool, but with medium-sized acidic fruits. The accessions included in this pool grouped in the previous AFLP analysis. Varieties of the *acidulus* group are currently grown as vegetables in India [29]; the third group included 5 genotypes of the exotic Far-East Asian variety, *conomon,* one of the most common source of resistances for cultivated melons, which is characterized by medium-sized, climacteric or non climacteric fruits, with variable fruit quality traits. This group includes typical var. *conomon* as well as others belonging to the varieties *chinensis* and *makuwa*. Varieties of these groups are still widely cultivated as vegetables in rural areas of China [30]. The *conomon* group was represented by 2 genotypes in the previous Sanger and 454 massive sequencing assay [27], and includes the accession Songwhan Charmi, one of the parental lines of the melon genetic map and of the DHL used for whole genome sequencing [28,31]. The fourth RNA pool included 7 representatives of three varieties that have been previously classified in the subsp. *melo* (*dudaim* and *flexuosus*) and *agrestis* (*momordica*), but are often considered intermediate between the two subspecies based on molecular studies [9,11,32]. This group includes cultivated snake melons consumed immature as cucumbers in southern Europe, northern Africa, and the Middle East, one known oriental cultivar of mango melon used as an ornamental, and snap melon cultigens grown in India.

The remaining four pools represented the variability of the cultivated types of subsp. *melo* (Table 1): the fifth group included 8 *cantalupensis* commercial varieties and 5 *cantalupensis* breeding lines belonging to the Charentais market class from Semillas Fitó. This group comprises the botanical varieties *cantalupensis* and *reticulatus*, which include many economically important cultivars from Europe, Asia and America. Previous Sanger and 454 sequencing assays included 3 representatives of this group [27]; the sixth RNA pool was formed by 11 melon cultivars representing other melon varieties, i. e. *adana*, *chandalak*, and *ameri*, most of which show intermediate characteristics between the two main economically important groups, *cantalupensis* and *inodorus*, and several *inodorus* cultivars from Eastern Europe and Western and Central Asia; the seventh group was prepared from 15 Spanish cultivars of the *inodorus* group, including many market classes that are popular in Eastern and Southern Europe and Brazil (i.e., ‘Amarillo’, ‘Rochet’, and ‘Tendral’), as well as other less know types representing the variability of the Spanish melon landraces; the most important *inodorus* market class, Piel de Sapo, was resequenced in a separate group, which included the cultivar T111 and 5 additional breeding lines provided by Semillas Fitó. The cultivar T111 was included in the previous massive sequencing assay, and is the parental of the genetic map of melon [27].

### cDNA preparation and sequencing

Total RNA was isolated from leaf tissue using the Trizol method in the 67 selected genotypes and stored at −80°C until library construction. Equivalent amounts of RNA from each genotype were combined into eight pools. mRNA was purified from the total RNA using the illustra^TM^ mRNA Purification Kit (GE Healthcare, Amersham Bioscience). Quantification and quality analysis was performed by agarose electrophoresis and by using Spectrophotometer NanoDrop ND-1000 v 3.5.

Double-stranded cDNA was then synthesized from the RNA pools with the SMART ^TM^ PCR cDNA Synthesis Kit (Clontech). cDNA PCR products were purified using the RocheÂ´s High Pure PCR Cleanup MicroKit and a subsequent precipitation with sodium acetate. Another quantification step using electrophoresis and spectophotometry was also carried out. A normalization step was carried out with the TRIMMER cDNA normalization Kit (Evrogen) in order to prevent over-representation of the most common transcripts. cDNA was amplified with the Advantage 2 PCR Kit (Clontech) in order to obtain the required quantity. The performance of the normalization step was checked by quantitative PCR with FastStart Universal SYBR Green Master (ROX) (Roche). Samples to be sequenced were lyophilized after purification and precipitation. Approximately 10 μg of double-stranded cDNA from each of the eight normalized cDNA pools were used for sequencing on a SOLiD v4 following standard procedures.

The Applied Biosystems SOLiD™ System uses the sequence-by-ligation technique to generate several gigabases of short sequence reads in a single run. Error rates are higher in comparison to those of Sanger sequencing reads, but the sequence-by-ligation technique takes advantage of a two-base encoding scheme to help identify these errors. Templated beads were prepared from each of the eight transcriptome libraries according to the manufacturer's instructions using the ePCR kit v.2 and the Bead Enrichment Kit from Applied Biosystems (Life Technologies, Inc.) for SOLiD3. Workflow Analysis was done after the first round of template bead preparation for each library according to the manufacturer's instructions using the Workflow Analysis kit from Applied Biosystems (Life Technologies, Inc.) to check library quality and the amount of templated beads generated per ePCR. An additional Workflow Analysis was done after it was estimated that a sufficient number of templated beads has been produced. Templated beads were deposited on slides according to the manufacturerÂ´s instructions using the Bead Deposition kit from Applied Biosystems (Life Technologies, Inc.). A 1/8 sequencing run was performed for each pooled transcriptome library (Sistemas Genomicos S.L).

### Read processing, mapping and SNV mining

Raw reads generated with SOLiD were processed using the ngs_backbone pipeline [33,34] with the configuration file included as Additional file 2 “ngs_backbone configuration”. Reads were cleaned by following the quality standards for SOLiD reads proposed by Sasson and Michael [35]. The sequences with more than two missing calls or with a mean quality lower than 15 in the first 10 bases were removed. The 3Â´ regions with a mean quality lower than 20 were trimmed to improve the mapping and the reads with a length below 30 were also dropped. A first draft of the entire melon genome sequence was recently developed under the framework of the MELONOMICS project (2009–2012) of the Fundación Genoma España [28]. This sequence was generated from the double haploid line DHL92 derived from the cross between Piel de Sapo T111 and the *conomon* variety Songwhan Charmi.

In order to make the best use of the short sequence reads for SNVs (Single variants: short INDELs and SNPs) discovery, processed SOLiD reads were aligned to this available melon genome assembly (v3.5) [36]. Alternatively the SNPs were also referred to the transcriptome available at http://melonge.net build with the reads described in Blanca et al. [27]. The method used to do this transcriptome based SNV calling was exactly the same as the described for the genome.

Reads were mapped using BWA [37] run with its default parameters. Other mappers capable of dealing with the splice junctions were assessed like TopHat. TopHat failed to create valid SOLiD mapping with the version available at the time. Several sets of BWA parameters were tested and found to map more reads, but they were dismissed because they were less stringent than the default ones. The SNVs were called with ngs_backbone. Stringent criteria for the SNV calling were used, and only those regions with at least 6X coverage were mined for SNVs. The SNVs were required to have a quality of 70 and at least 3 reads per allele. The obtained SNVs were filtered to select those that were variable within and among groups and to facilitate its use in high-throughput genotyping platforms [27]. The configuration of the filters can be also found in the nsg_backbone configuration file included in Additional file 2.

## Results and discussion

### Sequence generation, processing and mapping

The 8 pooled libraries were sequenced separately in one SOLiD run, generating a total of 260 million (M) reads of 49-bp (12.737 Gb of sequence). These reads were deposited in the NCBI Sequence Read Archive (SRA) with code SRA050003.2. An average of 32 M reads was generated per library. After cleaning with ngs_backbone, a total of 150 M reads were obtained with an average length of 44 bp, comprising 6.654 Gb. The total yield of sequences per pool was variable, ranging from 8.4 to 30.6 M, with the *melos* (pool 6) and African *agrestis* (pool 1) groups retaining the lowest and the highest numbers of useful sequences, respectively. Pool 6 was the one with the lowest sequencing quality. Changes in read number and average quality after read cleaning are detailed in Additional file 3: “Changes in number and quality of reads after processing with ngs_backbone”.

The cleaned reads were mapped by BWA [37]. About 50% of the reads, a total of 73 M (Table 1), could be mapped against the reference melon genome and used for SNV calling. The reference genome assembly consists of approximately 375 Mb arranged into 78 primary scaffolds, which represent 90% of the assembly, plus several thousand additional scaffolds and contigs [28]. The melon genome assembly can be accessed from the MELONOMICS webpage [36]. The cleaned reads were also mapped against the new version of the reference melon transcriptome of 49,741 unigenes available at http://melogene.net.

### SNP calling, number, and distribution

We identified a large number of genetic variants across the transcriptomes. A total of 303,883 SNVs, including SNPs and INDELs, were detected. Information about this SNVs collection is included in Additional file 4: “SNVs detected by mapping SOLiD sequences against melon genome”. This number is at least 7 fold higher than that identified previously by the Sanger and 454 sequencing of 10 representatives of 3 botanical varieties (38,587 SNPs and 5,795 INDELs) [27].

Information about the 239,521 SNVs identified by mapping SOLiD reads against the reference transcriptome instead of the genome is also included in Additional file 5 “SNVs detected by mapping SOLiD sequences against melon transcriptome” and can be accessed in http://melogene.net.

SNVs were distributed in 245 different scaffolds and contigs of the reference genome. Most (283,206, 93%) were located in annotated genes. The list of SNVs located in annotated genes is included in Additional file 6. “Location of SNVs in melon genes.”

The annotation of the newly assembled genome predicted 27,427 protein-coding genes, 15,064 of which contained variants, with an average of 18.8 SNVs per gene. 65.7% of the detected variants in genes were in CDS and the remainder in UTRs, with the UTRs displaying a higher SNV density, 14.9 SNVs/Kb, than in the ORF, 9.5/Kb.

The errors that occur in SNVs discovery when using massive sequencing technologies have several major causes: (1) PCR artifacts, (2) sequencing errors, and (3) errors in the mapping of short reads to the reference sequence. You et al. [19], after comparing the 3 most popular SGS platforms, 454, Solexa, and SOLiD, found that INDEL errors accounted for most sequencing errors, mainly in 454 and SOLiD, with base substitution error rates being less frequent. The SOLiD platform exhibited the lowest base substitution error rate, likely reflecting the di-base encoding and color space scheme in this sequencing technology. Since INDELs are a significant source of false-positive variants, we filtered them out (filter VKS in Additional file 4). To compare the variability of the different groups, all short INDELs were excluded, and only high-quality SNPs were retained.

A 93% (283,972) of the SNVs detected by mapping SOLiD reads against the melon genome were SNPs. 94% (266,130) were located in annotated genes of the melon genome, distributed in UTRs (28.4%) and ORFs (67.6%), with an average density of 13.3 SNPs/Kb *versus* 9.3 SNPs/Kb, respectively. Due to the mapping procedure used, we did not identify any SNPs in intron-exon junctions. Further analysis of these regions would increase the total number of SNPs in the collection.

**Figure 1:**
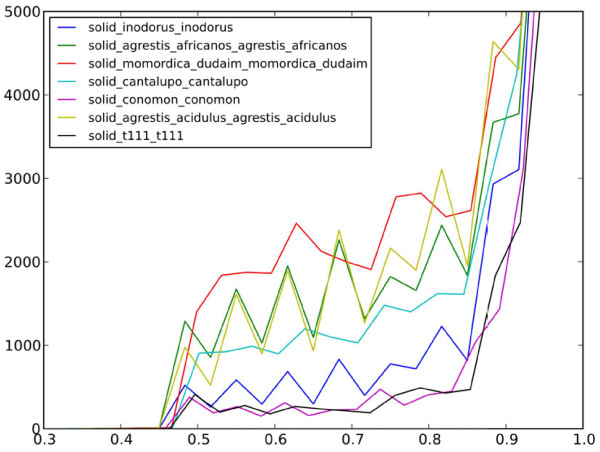
**MAF distribution of SNPs selected in the different sequenced pools.** The number of SNPs with different MAF values is represented for each pool.

For each SNP, the major allele frequency (MAF) was estimated from the available sequences. The proportion of SNPs with MAF <0.9 was 25.94%. Figure 1 shows the MAF distribution of SNPs detected in each pool.

This is the largest SNP collection available in *C. melo* to date. A collection of several thousand SNPs (about 3,000) was reported, generated from a much more limited collection of sequences obtained using traditional Sanger methods [20,21]. Massive sequencing has only very recently been applied to melons, and has produced the first massive SNP collection, with a total of 38,587 SNPs, detected in the first combined transcriptome assembly with the Sanger and newly produced 454 sequences [27]. This previous study used a range of melon genotypes (10) representing two cultivated varieties of the subspecies *melo*, var. *inodorus* (including the Piel de Sapo market class) and var. *cantalupensis*, and the *conomon* variety of the subspecies *agrestis*. Blanca et al. [27] reported considerably lower SNP densities, from 0.2 to 1.5 SNPs/Kb. The two results are difficult to compare as the coverage and the number of varieties represented are higher in this study. However, we consider that the higher number of SNPs reported here is mainly due to the high number of materials included in the study, as the more diverse the materials sequenced the more variation is sampled. The SNP density found in this study is more similar to that reported after the resequencing of the transcriptomes of several genotypes in several other crops sequenced mostly by means of 454 and Solexa [13,38,39], but none of these marker sets come from such a large germplasm collection. Much larger SNPs collections, with several million SNPs, have been reported after the whole genome resequencing of several crop genotypes [19,40,41]. However, most of the reported SNPs are in non-genic regions, and the number and relative distribution in CDS and UTRs is comparable to the hundreds of thousands presented here.

### Within-group variation

Table 2 shows the total sequence length (with a minimum of 6X coverage) used for SNP mining in each pool, ranging from 4.4 Mb (pool 6, group *melo*) to 15.7 Mb (pool 4, group *momordica*). The number, density and variability of *in silico*-detected SNPs, varied among groups.

**Table 2:** **SNPs identified in the eight pools of *C. melo *genotypes resequenced with SOLiD**

**Pool**	**Sequence length**^**1**^	**Total N° SNPs**^**2**^	**SNPs/kb**	**N° SNPs with MAF < 0.7 (%)**^**3**^
*C. melo* subespecies *agrestis*				
1) African *agrestis*	13,230,637 bp	117,204	8.9	9,133 (7.8)
2) Asian *agrestis*-*acidulus*	14,275,353 bp	96,460	6.8	10,197 (10.6)
3) Far East *conomon*	13,218,638 bp	81,807	6.2	1,305 (1.6)
Intermediate types				
4) Middle East and Indian *momordica-dudaim-flexuosus*	15,745,206 bp	132,792	8.4	13,826 (10.1)
*C. melo* subespecies *melo*				
5) Group *cantalupensis*	13,982,666 bp	102,565	7.3	6,317 (6.2)
6) Group *melo* Europe-Asia *inodorus-chandalak-ameri*	4,430,082 bp	40,762	9.2	2,417 (5.9)
7) *inodorus* Spanish landraces	12,505,399 bp	79,551	6.4	3,210 (4.0)
8) *inodorus* group market class Piel de Sapo	8,680,064 bp	43,363	4.9	1,396 (3.2)

^1^ Number of nucleotides sequenced at least 6 times used for SNP mining in each pool.

^2^ Total number of SNPs detected within each pool (SNPs with two or more alleles within the corresponding group).

^3^ Total number of highly variable SNPs (those with a frequency of the major allele, MAF, <0.7). In parentheses the percentage over the total number of SNPs is indicated.

SNP densities in the pools with accessions belonging to the subspecies *agrestis* were similar to those of the subspecies *melo* (ranging from 4.9 to 9.2 SNPs/Kb). However, the percentage of highly variable SNPs (with MAF under 0.7) was higher in *agrestis* pools including wild and exotic accessions from Africa and Southern Asia (pools 1 and 2) (Figure 1). The level of molecular variability in these two pools was similar despite pool 2 was more heterogeneous (Table 1, Additional file 1). High variability in the *agrestis* and *acidulus* from these areas, which are putative centers of origin for melon, was previously reported [29,42,43]. Less variable were the *conomon* from the Far East (pool 3) even when the included accessions were quite phenotipically variable (Table 1; Additional file 1). In this group only 1.6% of the detected SNPs had MAF < 0.7, which is consistent with previous studies that found East Asian melons to be less variable than South Asian melons (especially those from India) [30,44-46].

In our study, pool 4 also showed a large SNP density and a high percentage of highly variable SNPs (>10%) (Figure 1, Table 2), which is consistent with the higher taxonomic variability of this pool composed of *momordica*, *dudaim* and *flexuosus* genotypes from India and the Near and Middle East (Table 1; Additional file 1). The *momordica* group has been reported to show high levels of genetic diversity [47-49]. In addition, high levels of variability, leading to discrepancies in their taxonomic classification, have been reported for *dudaim* and *flexuosus*, as accessions of these groups are sometimes grouped with *agrestis* types or interspersed with sweet cultivated types of the subspecies *melo*[9,11,32]. These data agree with previous studies that indicate a higher molecular variability in Africa and Central and Southern Asia, than in the extremes of melon distribution (the Mediterranean area and the Far East) (reviewed in Esteras et al. [2]).

The previously described pools, 1 to 4, mostly include non-sweet melons found growing wild or locally cultivated as exotic vegetables in different parts of the world. We present here for the first time a deep understanding of their genetic variation. This knowledge can be used to provide the basis not only for breeding commercial sweet melons (*cantalupensis* and *inodorus*), but also for promoting their own conservation and for starting commercial breeding activities for these exotic crops. In this sense, Fergany et al. [29] and Kong et al. [30] observe the need to develop new varieties with higher yields and improved nutritional value of *acidulus* and *conomon* melons, which are in high demand in India and China.

Unlike other crops for which a extremely narrow genetic basis is reported in cultivated material after resequencing, such as cereals [19], or tomato [50] some of the sweet melon groups still retain significant levels of diversity. The *cantalupensis* group (pool 5) (which includes melons of several market classes, Charentais, Galia, etc.) was the most variable, with MAF values similar to those of the *agrestis* group (Figure 1). All the sequenced cultivars are commercial cultivars subjected to breeding. The combination of genetic material from different groups by breeders or the introgressions of favorable traits from wild or exotic material during breeding programs may account for part of this variation. The other major commercial group (pool 8), which includes only the Piel de Sapo market class (the most economically important of the *inodorus* melons), was less variable, as expected. Despite this low variability, 3.2% (1,396) of the 43,363 SNPs detected in this group were highly informative with MAF < 0.7, and represent the largest set of SNPs detected for this group to date.

The *cantalupensis* and *inodorus* groups are thought to have originated from genotypes distributed in Eastern Europe and Western Asia. The current variability of landraces and local cultivars in this area, including Turkey, Iran, Iraq, Russia, Ukraine and surrounding countries has only started to be analyzed [51]. Sensoy et al. [52] found many intermediate forms between the *inodorus* and *cantalupensis* groups in Turkey due to the traditional farming practices employed by some local small-scale melon producers. Kohpayegani and Behbahani [53] reported high variability in Iranian melon, comparable to that of Turkish melons and much higher than landraces from Europe. Nimmakayala et al. [54] first reported high variability in the botanical varieties *ameri*, *adana* and *chandalack* from Ukraine, considered to be the ancestors of the *cantalupensis* group. Most of these groups of cultivars are represented in pool 6. Even though this highly heterogeneous group had the lowest percentage of mapped reads (Table 1), most likely caused by a low sequence quality, it displayed a considerable number of highly variable SNPs.

Today the variation of the *inodorus* group is maintained in groups of landraces in different Mediterranean countries such as Greece and Italy [47,55,56]. The Iberian Peninsula is considered to be a secondary diversification center for melon and is a major world producer of *inodorus* cultivars [57]. Several studies have analyzed the distinctive morphological characteristics of Spanish melon cultivars (texture and unique taste). Also a marked lack of gene introgression from other germplasm of diverse origin has been suggested using molecular markers [57,58]. We detected a considerable SNP density, 6.4 SNPs/Kb, within the selected group of landraces (pool 7) (different types of Cassaba melons) indicating that high levels of variation are still present in this traditional Spanish germplasm.

Variation found in these groups of cultigens and landraces (pools 6 and 7) might prove useful for breeding commercial melons.

### Variation among groups

**Figure 2:**
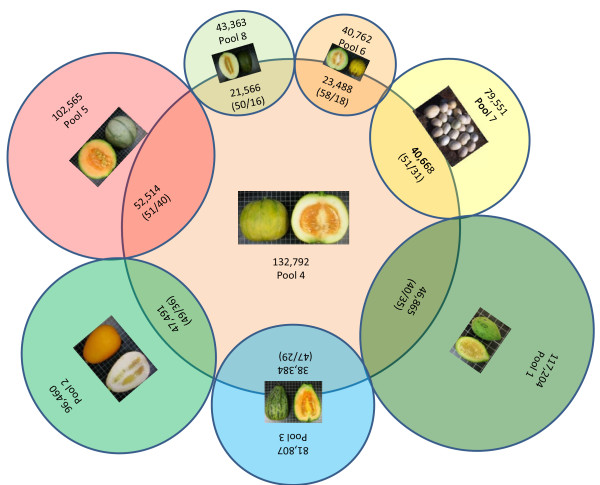
**Degree of shared polymorphism between the *momordica *group and the 7 pools of both subspecies.** Total number of SNPs in each group is indicated in the center of each circle and the number of shared SNPs in the intersection. Numbers in brackets show the percentage of shared SNPs (first number referred to the total number in each group and second number in the *momordica*).

Only 668 SNPs (0.2%) were shared among all libraries, with only 6 with MAF <0.7, which suggests the existence of differential variation in the different groups. Table 3 shows the amount of SNPs shared by every pair of libraries. The *momordica* group was the group with the highest percentage of SNPs in common with other libraries. Between 16 and 40% of the SNPs found in this group of exotic accessions were also variable in the commercial melons and landraces (Figure 2). The percentage of SNPs shared with exotic and wild *agrestis* was also high, ranging from 29 to 35%. The results are consistent with the intermediate position of the *momordica* group between both subspecies. The high heterogeneity of this pool might also explain this high level of shared variation with both subspecies, as it includes *flexuosus* and *dudaim* genotypes, which are often grouped with *agrestis* types, even though they have been reported to belong to subsp. *melo*[2]. Dhillon et al. [48] suggested that snap melon landraces from northern India might represent a central melon origin area from which oriental and occidental melon germplasm developed, a hypothesis that has also been supported by Luan et al. [46]. *Momordica* is one of the most utilized groups for melon breeding and serves to introgress resistance to pests and diseases and tolerance to abiotic stresses. These introgressions may also account for part of the shared variation.

**Table 3:** Number of SNPs shared and differential between groups

	**Piel de Sapo**	***Inodorus***	***Melo***	***Cantalupensis***	***Momordica***	***Conomon***	***Agrestis acidulus***	**African *agrestis***
	Pool8	Pool7	Pool6	Pool5	Pool4	Pool3	Pool2	Pool1
**Piel de Sapo**		15,560	9,149	19,451	21,566	14,168	14,488	18,172
Pool8		(36/20)	(21/22)	(45/19)	(50/16)	(33/17)	(33/15)	(42/16)
***inodorus***	1,564		16,255	33,966	40,668	25,165	26,914	32,236
Pool7			(20/40)	(43/33)	(51/31)	(32/31)	(34/28)	(41/26)
***melo***	3, 260	2,722		19,727	23488	13,541	15,589	17,157
Pool6				(19/48)	(58/18)	(33/17)	(38/16)	(42/15)
***cantalupensis***	4,735	4,353	3, 178		52,514	31,461	34,510	39,016
Pool5					(51/40)	(31/39)	(34/36)	(38/33)
***momordica***	4,441	4, 484	2, 417	4,224		38,384	47,491	46,865
Pool4						(47/29)	(49/36)	(40/35)
***conomon***	19,942	20,281	12,628	14,978	9,576		27,670	33,783
Pool3							(29/34)	(35/29)
***agrestis-acidulus***	11,402	12,009	6,837	9,577	5,273	6,474		36,162
Pool2								(44/31)
**African*agrestis***	20,501	20,931	14,132	18,294	13, 070	21,490	11,180	
Pool1								

Numbers in the upper half of the table indicate the number of common SNPs between each pair of libraries. Numbers in brackets indicate percentages these common SNPs represent of the total SNP set detected within the corresponding library (row/column). For example, there are 15,560 SNPs common between pools 8 and 7 (that is with two alleles or more in each of these pools). This number represents the 36% and 20% of the total SNPs detected within Pool 8 and pool 7 (indicated in Table 2) respectively.

Numbers in the lower part of the table indicate the SNPs that are fixed within each pair of libraries, but polymorphic between them. For example, there are 1,564 SNPs for which all reads from pool 8 have one allele and all reads from pool 7 have the alternative allele.

Despite the high level of shared variation, all the groups retained a number of exclusive SNPs. For example, 111,226 and 80,278 SNPs that were variable within the *momordica* group were not detected in Piel de Sapo and the *cantalupensis* commercial cultivarsrespectively. Table 3 shows the number of SNPs that differentiate pairs of libraries, i.e. nucleotide positions fixed within a given pool and different between pairs. The *momordica* group has thousands of fixed positions with different alleles in groups of subsp. *melo* (from 2,417 to 4,487), but this number is much higher in wild African (14,132 to 20,931) and even in Far Eastern *conomon* (12,628 to 20,218). These two groups were the most divergent from the subspecies *melo*. The largest differences were detected between *inodorus* and Piel de Sapo and the wild African *agrestis* group (over 20,501 SNPs). This suggests that a large portion of the genetic variability found within this melon collection has not yet been used for the development of new cultivars. Both, the African *agrestis* and *conomon* groups appear to represent essential reservoirs of underexploited variation. The large number of variants in which the two groups differ (21,490) suggests that they are rich complementary sources of genetic diversity for cultivated melons. The number of SNPs still present in the cultigens and landraces pools (6 and 7) that are absent from commercial cultivars (pools 5 and 8) are worthy of note as they may be useful for breeding melons using these sources that share similar genetic backgrounds.

### Variation in target genes

In order to validate the efficiency of this *in silico* SNP mining, we compared our results to those previously obtained using EcoTILLING in the same germplasm collection [59]. EcoTILLING was used to detect SNPs with an impact on gene function by screening the coding sequences of genes involved in fruit quality and disease resistance. The natural variation in two melon genes was analyzed: *Cm-ACO1* (1-aminocyclopropane-1-carboxylate oxidase 1) which is involved in melon ripening through the alteration of ethylene synthesis [60], and *Cm-eIF(iso)4E* (melon eukaryotic translation initiation factor E, Isoform) which has been suggested to be involved in recessive resistance to viruses [61,62]. In the previous study performed by Esteras et al. [59] all mutations found by EcoTILLING were confirmed by Sanger sequencing and the effect of the mutations was analyzed with SIFT (Sorting Intolerant from Tolerant) [63,64] which predicts whether an amino acid substitution affects protein function.

**Figure 3:**
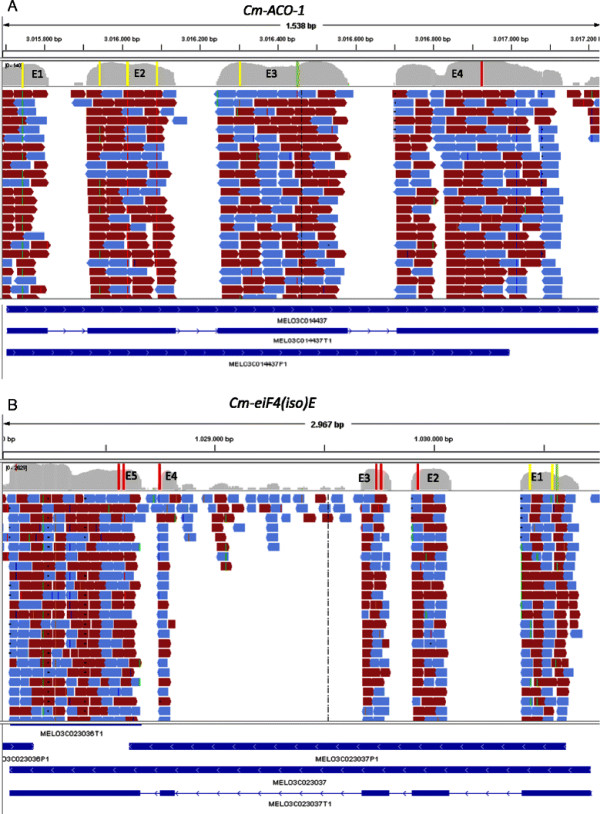
**SNPs detected in the coding region of *Cm-ACO-1 (A) *and *Cm-eiF(iso)4E (B). *** Short reads generated by SOLiD in the different pools are represented mapped to the genomic sequence (whole genome draft version 3.5 available in MELONOMICS) of both genes. Coverage in exonic and UTRs regions is shown for each nucleotide. SNPs detected by SOLiD and EcoTILLING are represented by colored bars in the different exons (red, green and yellow for mutations detected only by SOLiD, only by EcoTILLING and by both methods). The structure of each gene as annotated in the genome is shown below. Data are visualized with IGV (*Integrative Genomics Viewer)*[65].

*Cm-ACO-1* (unigene MELO3C014437 at [36]) is located in positions 3015704–3017224 of the scaffold CM3.5_scaffold00022 in the melon genome (v3.5) (Figure 3 A). Resequencing permitted us to find 6 SNPs in the coding region of this gene (Table 4). Five nucleotide variants were also previously detected by EcoTILLING [59]. The allele distribution found in SOLiD agrees with the EcoTILLING haplotypes: two mutations were exclusive to the *agrestis* pools (1, 2, and 3) (CM3.5_scaffold00022: 3015744 and 3016016), one was exclusive to the *conomon* pool (3) (CM3.5_scaffold00022: 3016091), and one was fixed in *agrestis* and appeared with a low frequency in the *momordica* and *melo* pools (4, 5, 6, 7 and 8) (CM3.5_scaffold00022: 3015944). According to EcoTILLING, the mutation CM3.5_scaffold00022: 3016304, the only predicted not to be tolerated by SIFT, was present in only one genotype, the snake melon from Arabia (included in pool 4, Table 1). Accordingly, the variant was only sequenced in pool 4, thus confirming the utility of pooling samples to increase the number of genotypes represented in resequencing assays without missing rare alleles.

**Table 4:** **Polymorphism in *Cm-ACO-1 *and *Cm-eiF4-iso *detected by SOLiD sequencing and EcoTILLING**[59]

**Position in the scaffold**^**1**^	**Position in the gene (from ATG)**	**SNP MAF**	**aa change**	**Effect on protein according to SIFT**^**2**^	**Exon**	**Detected by SOLiD/EcoTILLING**	***Agrestis***^**3**^	***Momord***	***Melo***
Coding region of *Cm-ACO-1*, CM3.5_scaffold00022									
3015744	41	A/G	D14G	Tolerated (1.00/3.02)	1	Yes/yes	G:6,7,4	A:21	A:5,2,8,-
		0.68							
3015944	241	A/G	L46L		2	Yes/yes	G:15,6,5	A:15	A:5,5,8,1
		0.54						G:1	G:1,0,1,0
3016016	313	T/G	L70L		2	Yes/yes	G:0,3,2	T:12	T:8,2,11,6
		0.99							
3016091	388	T/A	L95L		2	Yes/yes	T:3,1,0	T:10	T:7,-,2,2
		0.80					A:0,0,6		
3016304	601	C/A	L131M	Not tolerated	3	Yes/yes	C:8,4,6	C:12	C:9,4,20,5
		0.87		(0.03/3.02)				A:10	
-	747	C/T	D179D		3	No/yes			
3016920	1216	T/C	V294A	Tolerated	4	Yes/no	T:11,7,7	T:29	T:18,5,20,7
		0.97		(0.12/3.03)				C:1	C:1,0,0,1
Coding region of *Cm-eiF(iso)4E*, CM3.5_scaffold00057									
-	26	G/A	G9D	Not tolerated	1	No/yes			
				(0.00/4.32)*					
1030561	41	C/T	A14V	Not tolerated	1	Yes/yes	G:2,3,3	G:3	G:5,1,5,1
		0.90		(0.00/4.32)*			A:4,0,0		
1030440	162	T/C	L54L		1	Yes/yes	G:31,22,3	G:12	G:31,1,5,0
		0.70					T:0,1,0	T:1	T:3,3,21,7
1029938	664	C/T	S112N	Tolerated	2	Yes/no	C:66,33,26	C:25	C:0,15,55,19
		0.99		(0.56/3.11)			T:2,0,0		T:4,0,1,0
1029710	892	A/G	L153L		3	Yes/no	A:91,49,30	A:46	A:75,17,54,30
		0.99					G:1,0,0		G:1,1,0,0
1029697	905	C/T	K158E	Not tolerated	3	Yes/no	T:0,38,13	T:31	T:67,10,39,26
		0.99		(0.02/3.12)			C:5,0,0		C:3,0,0,0
1028781	1810	C/T	D178G	Tolerated	4	Yes/no	T:0,23,17	T:17	T:57,17,27,26
		0.99		(0.65/3.12)			C:7,0,0		C:1,0,2,0
1028629	1962	C/T	K198K		5	Yes/no	T:33,24,26	T:34	T:34,4,35,17
		0.99							C:2,0,1,0
1028619	1972	C/T	S202G	Not tolerated	5	Yes/no	T:42,32,33	T:37	T:52,8,49,21
		0.99		(0.00/3.14)				C:2	C:1,0,0,0

^1^ Position in the melon genome assembly v3.5 available at MELONOMICS [36].

^2^ The effect of mutations was analyzed with SIFT [64]. Prediction score and median sequence conservation, respectively, are indicated in brackets. * Low confidence in the prediction (few sequences represented at those positions).

^3^ Number of reads of each allele are indicated in each pool, (−) means that this nucleotide has not been sequenced in the corresponding pool, numbers are ordered according to pool number, *agrestis* (pools 1, 2 and 3), *momord* (pool 4), *melo* (pools 5, 6, 7, 8).

EcoTILLING studies show that most natural variation in *Cm-ACO-1* occurs in exon 1, 2 and 3 [59]. The only variant in exon 4 was detected by TILLING in an EMS-treated Piel de Sapo melon collection (C728T, T243I) [62]. SOLiD resequencing detected a putative natural missense mutation in exon 4, which was reported to be tolerated by SIFT (CM3.5_scaffold00022: 3016920). This was a rare allele (MAF = 0.97), only present in *momordica* and the two groups with commercial varieties, *cantalupensis* and Piel de Sapo. It has been demonstrated that two artificially induced missense mutations found in exon 3 (in a TILLING platform constructed in a *cantalupensis* genetic background) (C580T, L124Phe, and G791A, Gly194Asp) [66] delay the ripening process resulting in fruit flesh with increased firmness. It remains to be demonstrated if any of the natural putative missense mutations found in this study affect ethylene production, thereby altering the ripening process.

*Cm-eiF(iso)4E* (unigene MELO3C023037 at [36]) is located in CM3.5_scaffold00057: 1028066 to 1030714 (Figure 3 B). We detected 8 mutations in the coding region of this gene (Table 4). We previously screened the natural variation of this gene with EcoTILLING, analyzing exons 1, 2, and 3, and detecting only 2 of the 5 mutations identified by sequencing, both in exon 1 (CM3.5_scaffold00057: 1030561 and 1030440). Resequencing provided additional putative mutations in exons 2 and 3, one of which was non-tolerated. All were rare alleles that appeared in African *agrestis* accessions and in certain commercial varieties (CM3.5_scaffold00057: 1029938, 1029710, and 1029697). Exons 1, 2, and 3 of *Cm-eIF(iso)4E* were also tilled in the Piel de Sapo and Charentais TILLING populations described above [62]. Only one mutation in exon 1, a transition G128A that alters aa 43 R to K, was found and predicted to be tolerated, so the number of natural variants was much higher than that obtained with induced variation.

In the re-sequencing assay we also analyzed exons 4 and 5, which have not been analyzed by EcoTILLING. We found 3 rare mutations in *agrestis*, *momordica* and commercial cultivars respectively, the last of which was predicted to alter protein function according to SIFT (CM3.5_scaffold00057: 1028781, 1028629, and 1028619).

Although it is necessary to validate by sequencing or genotyping these *in silico-*detected SNPs, our results confirm that resequencing strategy provides a large catalog of alleles in genes of interest, some of which may potentially alter gene function.

Only two of the mutations detected by EcoTILLING in the accessions used for resequencing were missed by SOLiD: one in the *Cm-ACO-1* gene, mutation C/T in nucleotide 747 from the ATG, and the second in *Cm-eIF(iso)4E,* mutation G/A in nucleotide 26 from the ATG, both detected in the Wild chibbar accession of pool 2. Problems with the sequencing of the cDNA of this accession may explain these results.

### Design of a genotyping array for validation

To validate some of the putative SNPs found by resequencing we designed a Sequenom genotyping array [67] with 143 SNPs and used it with 78 varieties, including most of the resequenced genotypes (Additional file 7: “Validation of SNP”). To facilitate primer design and optimize the use of this genotyping method, the set of SNPs selected for validation was filtered out using IS60 and CS60 filters (see Additional file 4). These filters allow the selection of those SNPs that are not closer than 60 bp to an intron (193,743 SNPs, 68.2% of the total) or to another SNVs (55,000, 19.4%), respectively. CS60 was a very restrictive filter due to the large number of SNPs detected in the species, as only 19.4% of the detected variants donÂ´t have another SNVs in a flanking window of 60 pb, with only 28,996 (10.2%) meeting both criteria (no IS60 and no CS60). In order to increase the possibility of selecting SNPs that are useful for high-throughput genotyping, we modified filter CS60 to include those SNPs surrounded by SNPs with a very high MAF in the selection, that is, we allowed rare variants to be close to the SNPs assayed. The filter CS60_MAF permitted the selection of SNPs flanked by other SNPs with MAF values over a specified threshold. Table 5 shows the number of SNPs obtained after filtering the whole collection with different filter combinations. For example, the number of selected SNPs increased from 28,996 to 65,500 when we combined no IS60 and no CS60_MAF0.99. Only a small proportion of these SNPs were common to all resequenced groups.

**Table 5:** Number of SNPs meeting different criteria for optimizing validation with the sequenom genotyping array

**No CS60_MAF**^**1**^	**Whole collection**	**No IS60**	**Variable in all groups**
MAF 1	55,000	28,996	9
		(10.21%)	
MAF 0.99	108,731	65,500	158
		(23.07%)	
MAF 0.98	136,694	86,103	211
		(30.32%)	
MAF 0.97	150,590	96,657	231
		(34,04%)	
MAF 0.96	160,231	103,976	260
		(36,61%)	
MAF 0.95	167,718	109,734	277
		(38,64%)	
MAF 0.7	178,107	168,726	431
		(59,42%)	

^1^ Those SNPs having the filter CS60_MAF in Additional file 4 are flanked in a window of 60 bp by other SNPs with values of MAF over the threshold indicated. Columns indicate the number of these SNPs in the whole collection, in the subset free from introns in a flanking window of 60 bp (no IS60), and in the subset variable in all groups. These filters for each SNP of the whole collection are included in Additional file 4.

Using the subset of SNPs with no IS60 and no CS60_MAF, we randomly selected several sets of SNPs that met different within- and between-group variation criteria for validation. The number of SNPs selected from each group and the validation percentage is included in Table 6. All the assayed SNPs amplified in most samples and only 12 were monomorphic in all the accessions genotyped, giving a validation ratio of 92%. Similar validation rates have been previously reported with SOLiD and Solexa [19].

**Table 6:** SNPs variable within and between different groups of botanical varieties selected for validation

**Polymorphic between **^**1**^		**Total SNPs**			**SNPs with MAF <0.7**			**Selected**	**Validated**
		**All**	**No Is60**		**All**	**No Is60**			
**Monomorphic Within**	**Monomorphic Within**		**CS60 MAF1**	**CS60 MAF0.7**		**CS60 MAF1**	**CS60 MAF0.7**		
Piel de Sapo (8) *African agrestis* (1)	*conomon* (3)	13,168	4,000	6,240	5,361	1,659	2,690	34	33 (97%)
Piel de Sapo (8) *conomon* (3)	African *agrestis* (1)	15,261	4,226	7,095	6,724	1,894	3,322	24	24 (100%)
*cantalupensis* (5) *conomon* (3)	African *agrestis* (1)	13,168	3,559	5,972	5,052	1,354	2,284	12	12 (100%)
*momordica* (4) Piel de Sapo (8) *inodorus* (7)	African *agrestis* (1)*conomon* (3)	5,822	1,739	2,265	2,848	879	1,139	15	14 (93%)
*momordica* (4) *cantalupensis* (5)	African *agrestis* (1)*conomon* (3)	5,102	1,544	2,006	2,305	744	954	24	24 (100%)
Polymorphic in Piel de Sapo		43,363			1,305			19	12 (63%)
Polymorphic in all groups		668	9	431	3	0	0	16	13 (81%)

^1^ Pool number indicated.

The ratio of validation varied among SNPs groups. Nearly 100% of the SNPs selected for being common between Piel de sapo and African *agrestis* or *conomon*, and variable with *conomon* or African *agrestis*, respectively, were successfully validated (Table 6, and Additional file 7). Nearly all the SNPs selected for being common between *cantalupensis* and *conomon* and variable with African *agrestis,* and those selected for being common between *momordica* and *inodorus*-Piel de Sapo or *cantalupensis* and variable with *conomon* were also true SNPs. The percentage of validation was lower in the group of SNPs selected for being variable in all groups (81%), and the lower percentage of validation was found in the group variable within Piel de Sapo. However, the lower ratio of validation found in the latter group can be due to the fact that only 2 genotypes of this market class were included in the genotyping array due to technical problems.

Polymorphism Information Content (PIC) for every SNP validated was calculated by using Power Marker v. software [68] (Additional file 7). In general, results indicate a high percentage of validation and consistency of the results obtained by SOLiD with those of the genotyping array, suggesting that most of the *in silico* selected markers will be useful for different melon breeding objectives.

## Conclusions

This study provides the first comprehensive resequencing data of wild, exotic, and cultivated melons. It demonstrates that pooling RNA samples from several genotypes combined with high-throughput transcriptome sequencing is an efficient and effective way to identify large numbers of SNPs. This collection of variants dramatically improves the previously available SNP collection by increasing the total number of useful SNPs and by identifying new ones in groups of melons from the area of origin and diversification analyzed here for the first time. Our results show the divergence between wild and cultivated melons. The huge amount of variation present in wild African *agrestis* and *conomon,* which is absent in the subspecies *melo,* may prove useful in breeding commercial types. The variation detected in landraces shows that these are also reservoirs of polymorphism for breeding melons with similar genetic backgrounds. The high percentage of validation confirms the utility of the SNP-mining process and the stringent quality criteria for distinguishing sequence variations from sequencing errors and mutations introduced during the cDNA synthesis step. The availability of this information will aid in carrying out future studies of population genetics, marker-assisted breeding, and QTL dissection. Some of the resequenced genotypes are donors of agronomic traits, with available mapping population’s with will enable the rapid application of the discovered SNPs in mapping experiments.

## Competing interests

The authors declare that they have no competing interests.

## Authors' contributions

BP, JB and JC were involved in the conception and design of the study. BP provided the melon core collection and selected the genotypes for sequencing. CE, CR and JC prepared the normalized cDNA libraries for sequencing. VF-P, CC and RR were involved in the sequencing of normalized cDNA libraries in SOLiD platform: construction of SOLiD barcoded libraries from cDNA, pooling of the libraries, emulsion PCR and sequencing in SOLiD 4.0., and AB was involved in coordination activities related with sequencing throughout the project. JB, JC, PZ and DP conducted the bioinformatic analysis, reads processing, SNP mining and mapping to the melon genome and trsnacriptome. BP selected the SNPs and genotypes for validation. CE, CR and BP validated the SNPs. CE and BP performed EcoTILLING and analyzed mutations. BP was primarily responsible for drafting and revising the manuscript with contributions from co-authors. All authors read and approved the final manuscript.

## Supplementary Material

Additional file 1 **Resequenced melon genotypes.** Photographs of the fruits of the genotypes resequenced, in eight pools, using SOLiD are included. **A**. Pools 1–4. **B**. Pools 5–8. Click here for file

Additional file 2 The configuration of the ngs_backbone pipeline used for processing raw reads generated with SOLiD, for mapping, SNV calling and filtering is included. Click here for file

Additional file 3 Changes in number and quality of reads after processing with ngs_backbone. Click here for file

Additional file 4 **SNVs detected by mapping SOLiD sequences against melon genome.** All SNVs detected in all eight resequenced pools are included, their position in the reference genome (scaffold or contig), referred to the whole genome draft version 3.5 available in MELONOMICS [36], their MAFs and allelic frequency in each group, and the filters implemented for its selection are detailed. Click here for file

Additional file 5 **SNVs detected by mapping SOLiD sequences against melon transcriptome.** All SNVs detected in all eight resequenced pools are included, their position in the reference transcriptome available in http://melogene.net, their allelic frequency in each group are detailed. Alleles in reads from genotypes previously sequenced with Sanger and 454 are also indicated. Click here for file

Additional file 6 **Location of SNVs in melon genes.** Correspondence of the SNVs located in melon genes annotated in the melon genome version 3.5 available in MELONOMICS [36] is listed. Click here for file

Additional file 7 **Validation of SNPs.** Information about the SNPs selected for validation is included along with genotyping results obtained with Sequenom with 78 varieties. PIC for each SNP along with the MAF estimated by SOLiD and by genotyping is indicated. Click here for file
