# Supplementary material for: Transcriptome sequencing for SNP discovery across Cucumis melo
Source: BMC Genomics. 2012 Jun 24;13:280. doi: 10.1186/1471-2164-13-280 (PMC3473316; doi:10.1186/1471-2164-13-280)
Supplement: Additional file 1 — Resequenced melon genotypes. Photographs of the fruits of the genotypes resequenced, in eight pools, using SOLiD are included. A. Pools 1–4. B. Pools 5–8. [file 1471-2164-13-280-S1.ppt]

## Slide 1
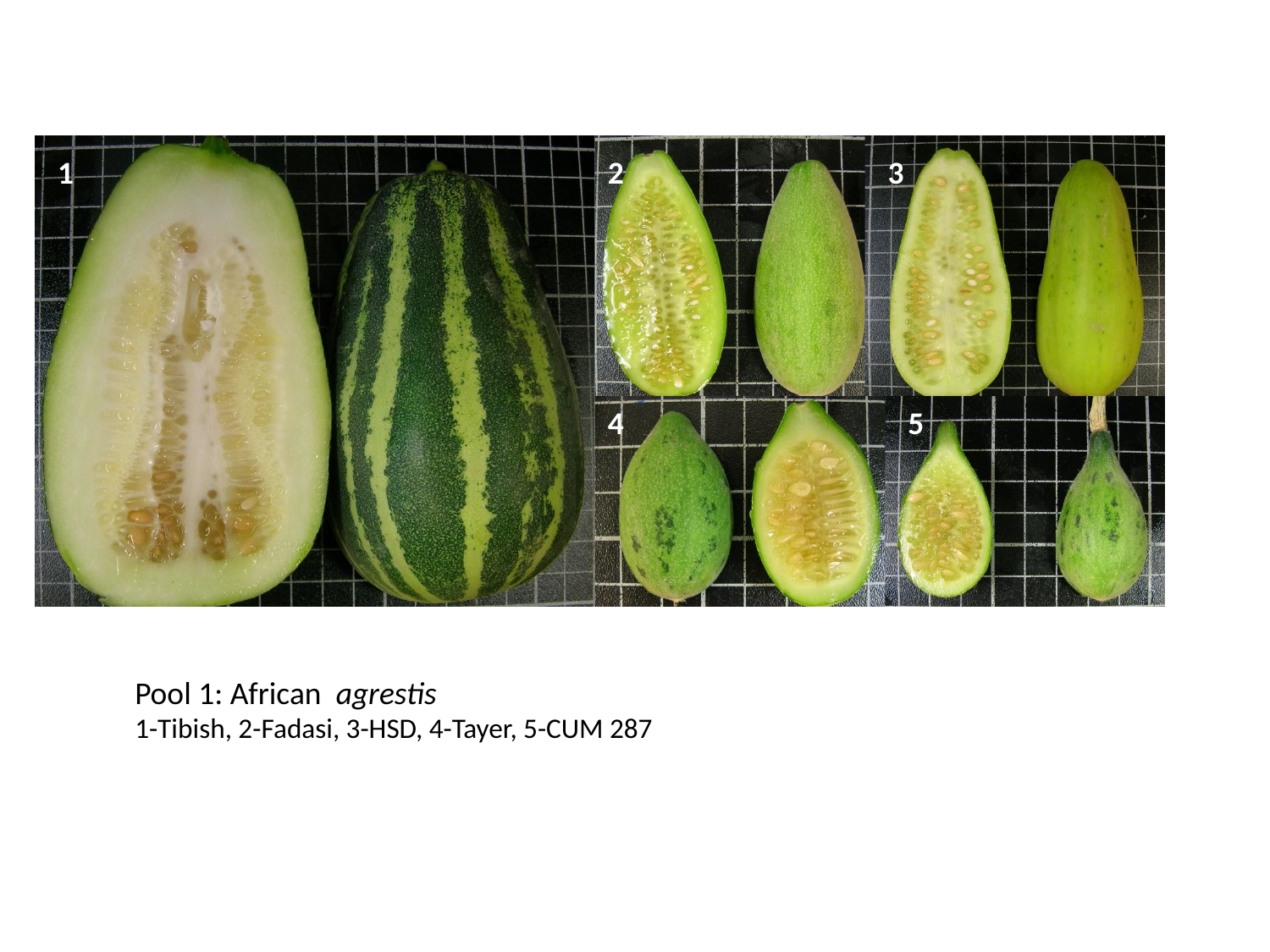

1
2
3
4
5
Pool 1: African agrestis
1-Tibish, 2-Fadasi, 3-HSD, 4-Tayer, 5-CUM 287

## Slide 2
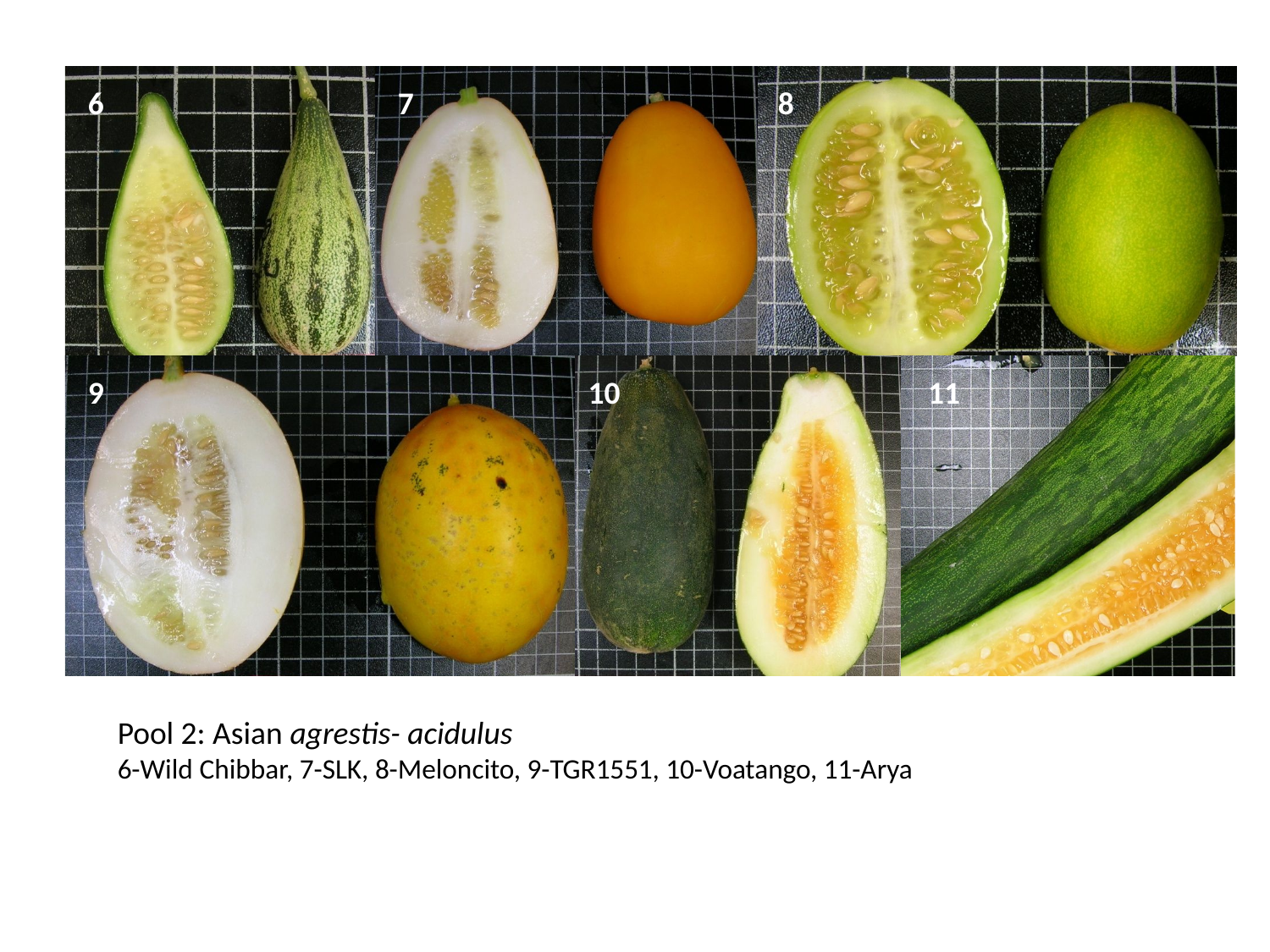

6
7
8
9
10
11
Pool 2: Asian agrestis- acidulus
6-Wild Chibbar, 7-SLK, 8-Meloncito, 9-TGR1551, 10-Voatango, 11-Arya

## Slide 3
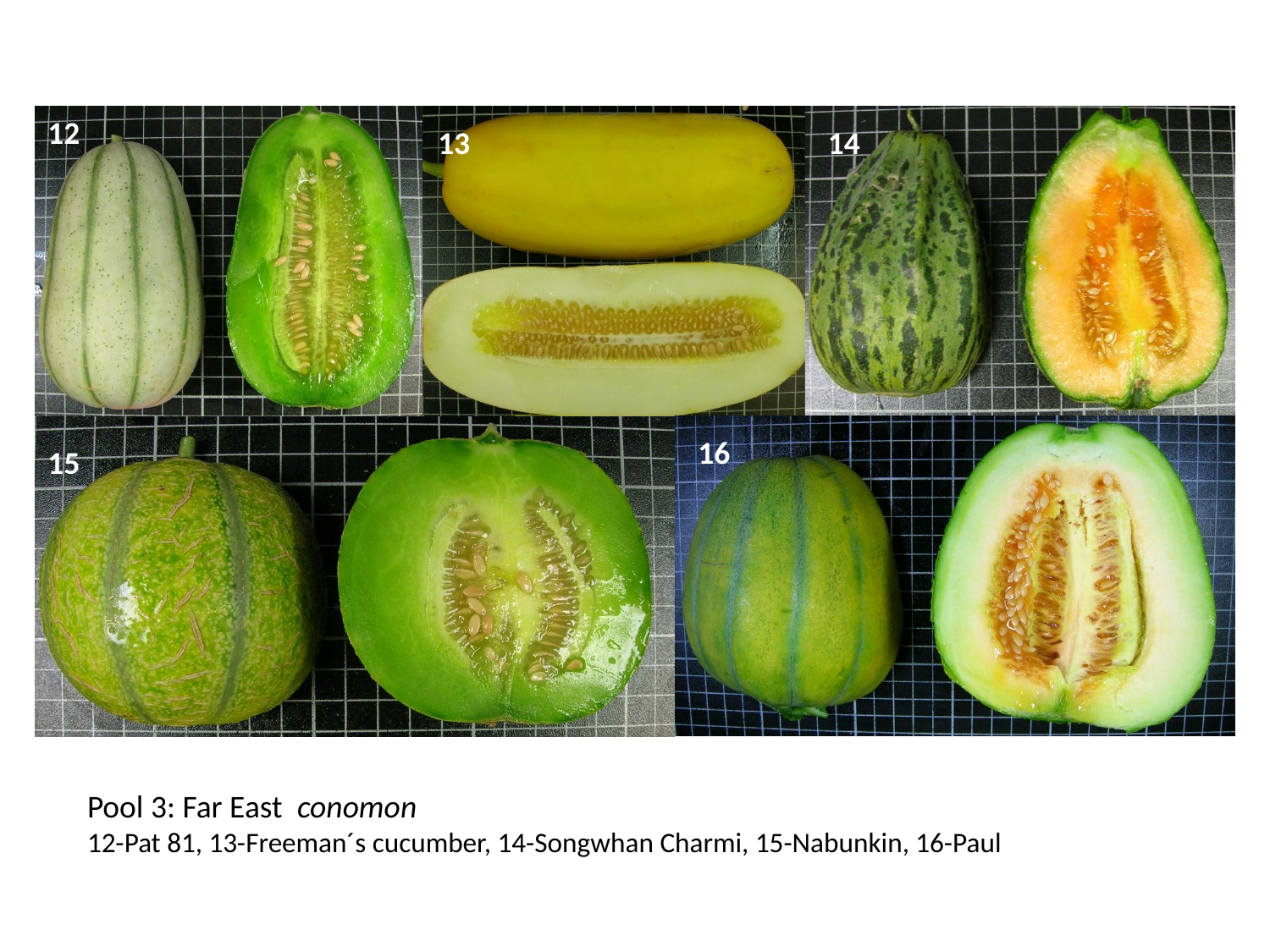

12
13
14
16
15
Pool 3: Far East conomon
12-Pat 81, 13-Freeman´s cucumber, 14-Songwhan Charmi, 15-Nabunkin, 16-Paul

## Slide 4
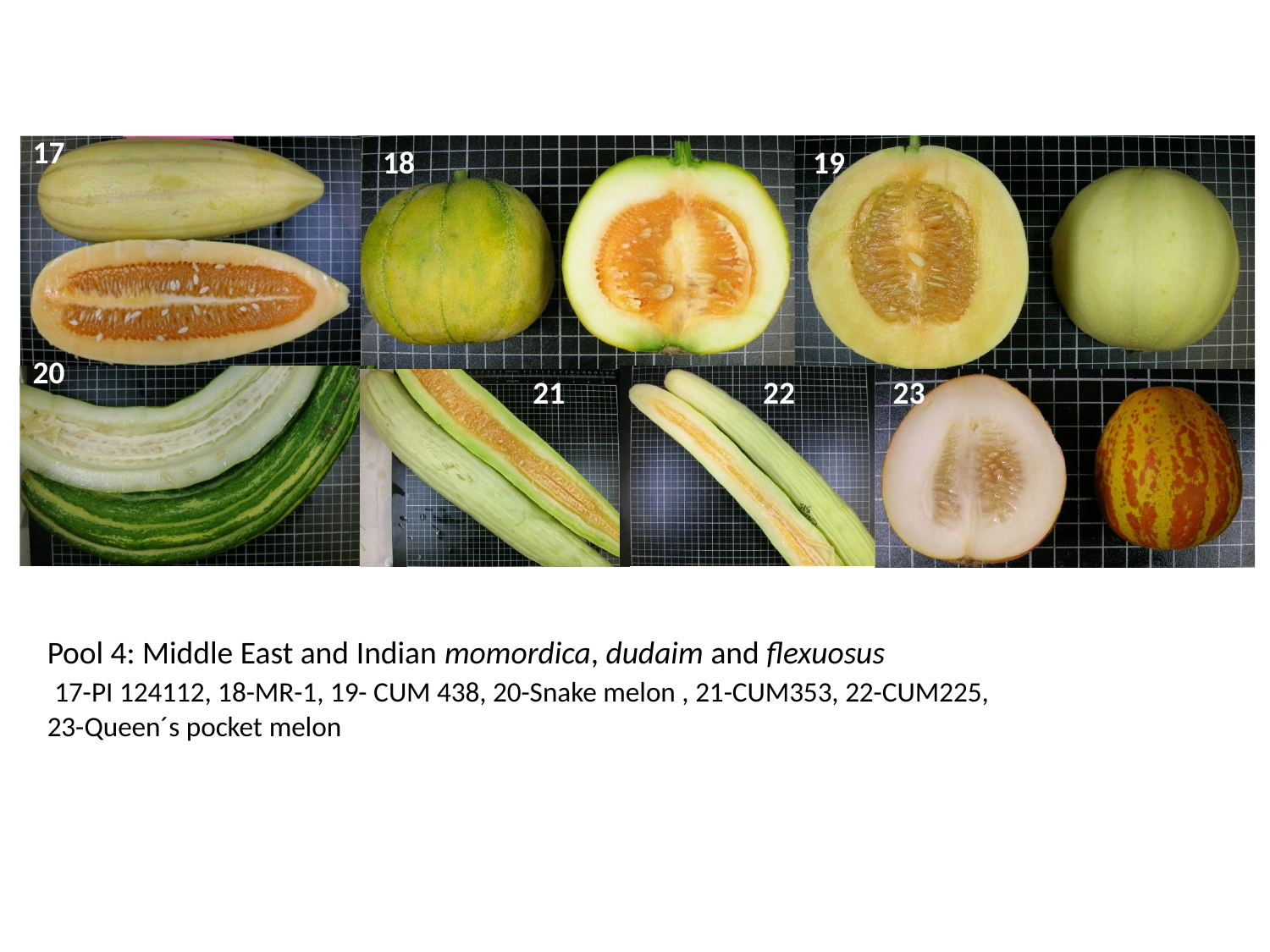

17
18
19
20
21
22
23
Pool 4: Middle East and Indian momordica, dudaim and flexuosus
 17-PI 124112, 18-MR-1, 19- CUM 438, 20-Snake melon , 21-CUM353, 22-CUM225, 23-Queen´s pocket melon

## Slide 5
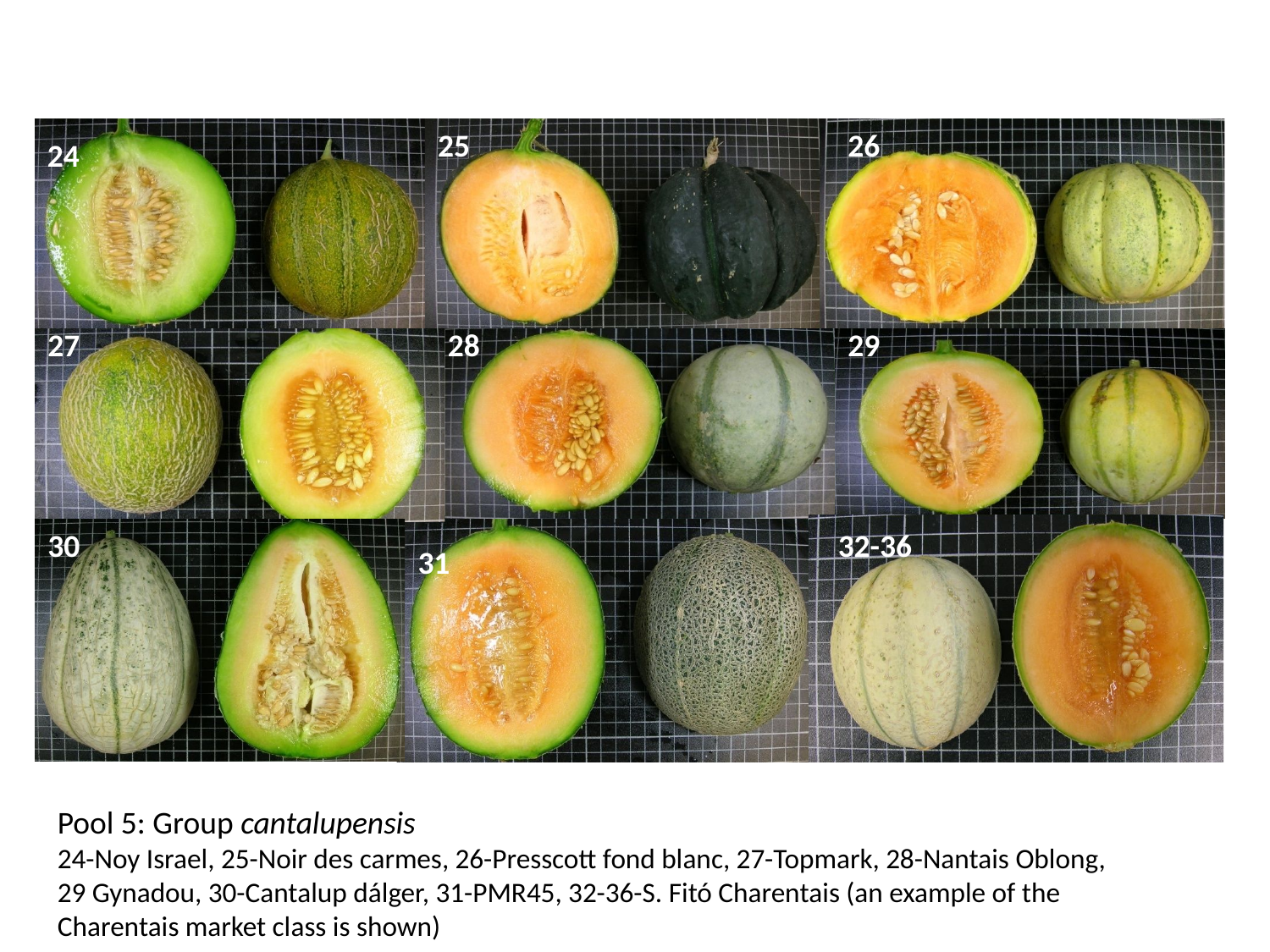

25
26
24
27
28
29
30
32-36
31
Pool 5: Group cantalupensis
24-Noy Israel, 25-Noir des carmes, 26-Presscott fond blanc, 27-Topmark, 28-Nantais Oblong, 29 Gynadou, 30-Cantalup dálger, 31-PMR45, 32-36-S. Fitó Charentais (an example of the Charentais market class is shown)

## Slide 6
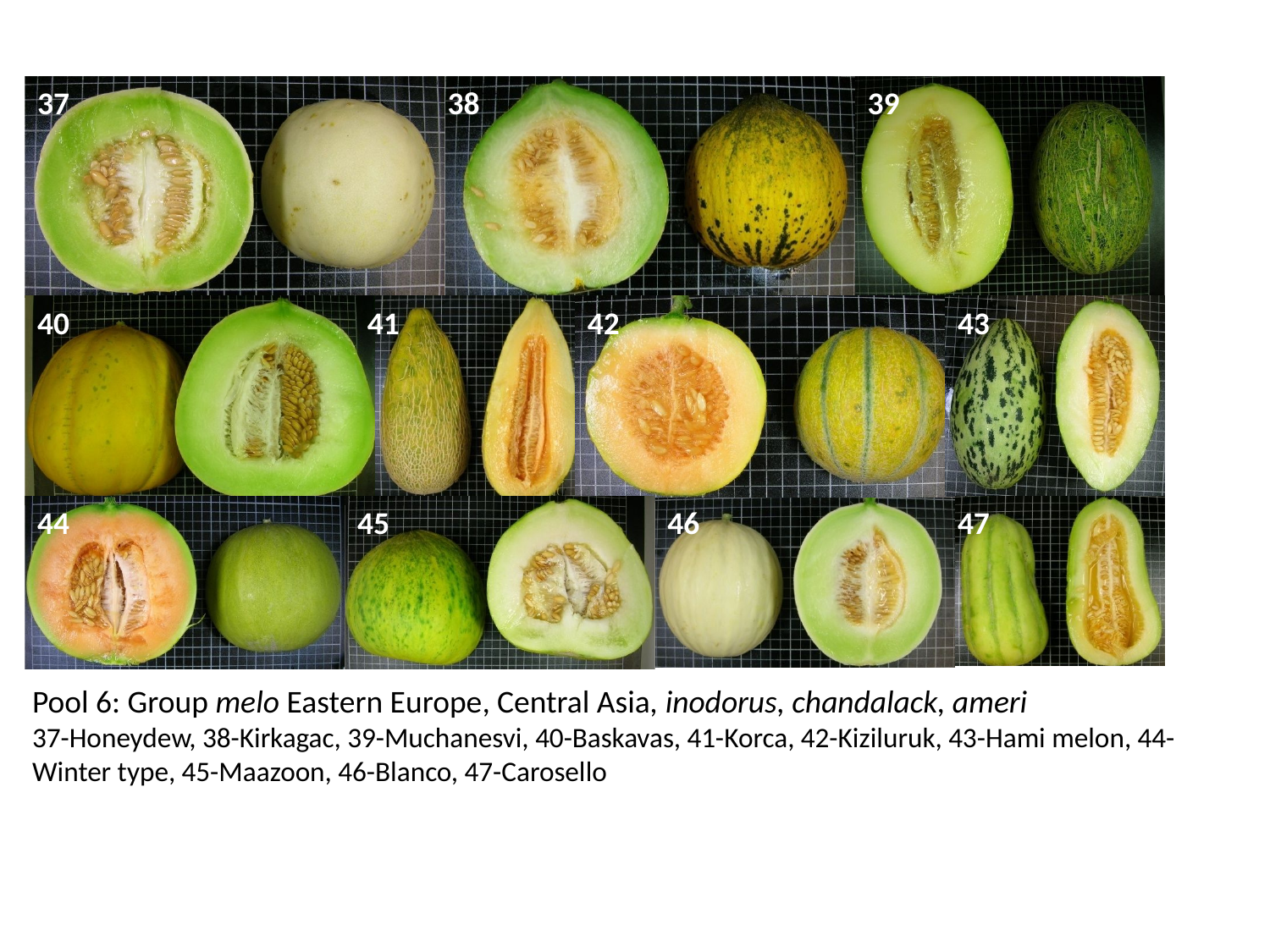

37
38
39
40
41
42
43
44
45
46
47
Pool 6: Group melo Eastern Europe, Central Asia, inodorus, chandalack, ameri
37-Honeydew, 38-Kirkagac, 39-Muchanesvi, 40-Baskavas, 41-Korca, 42-Kiziluruk, 43-Hami melon, 44-Winter type, 45-Maazoon, 46-Blanco, 47-Carosello

## Slide 7
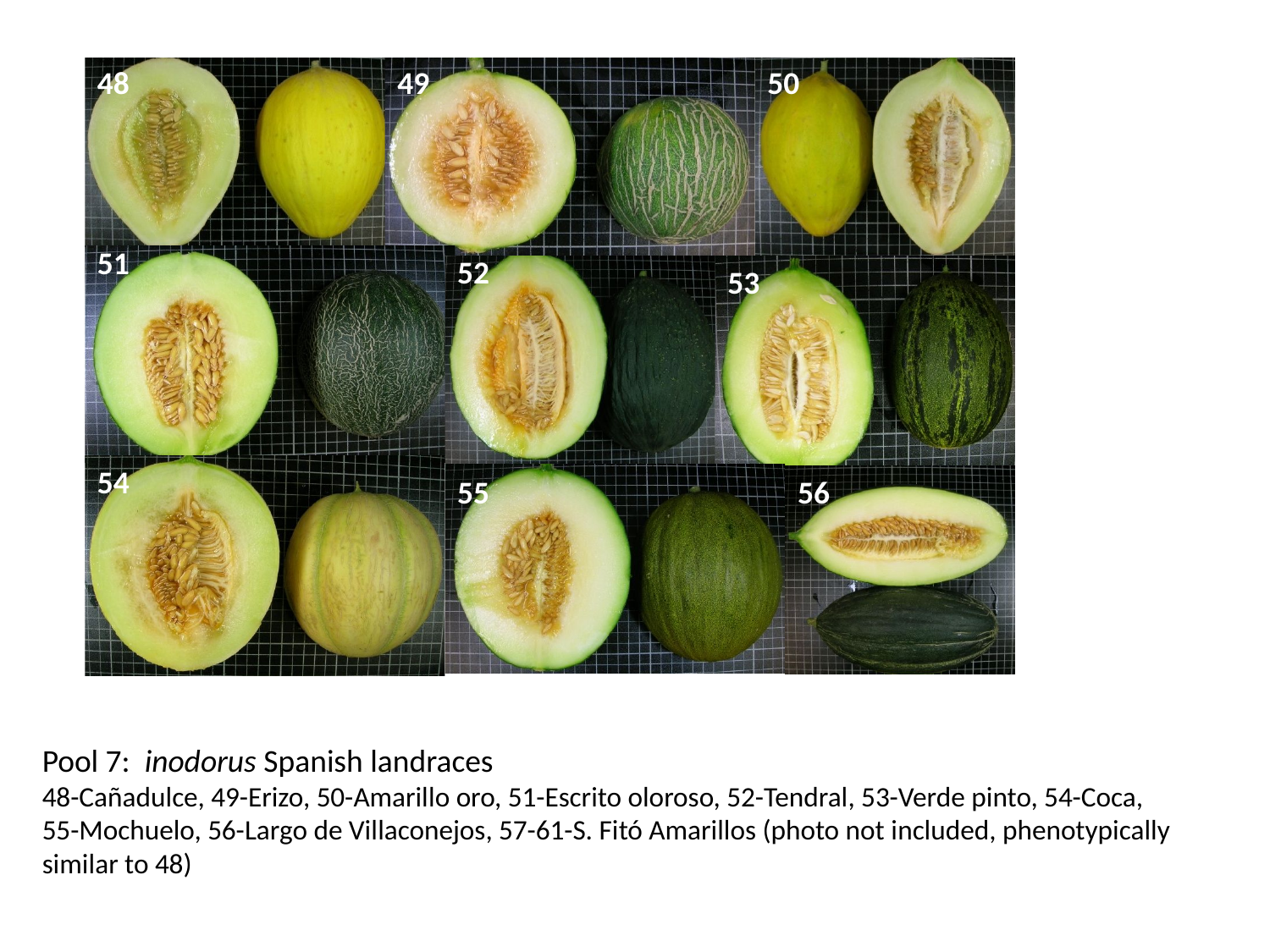

48
49
50
51
52
53
54
55
56
Pool 7: inodorus Spanish landraces
48-Cañadulce, 49-Erizo, 50-Amarillo oro, 51-Escrito oloroso, 52-Tendral, 53-Verde pinto, 54-Coca, 55-Mochuelo, 56-Largo de Villaconejos, 57-61-S. Fitó Amarillos (photo not included, phenotypically similar to 48)

## Slide 8
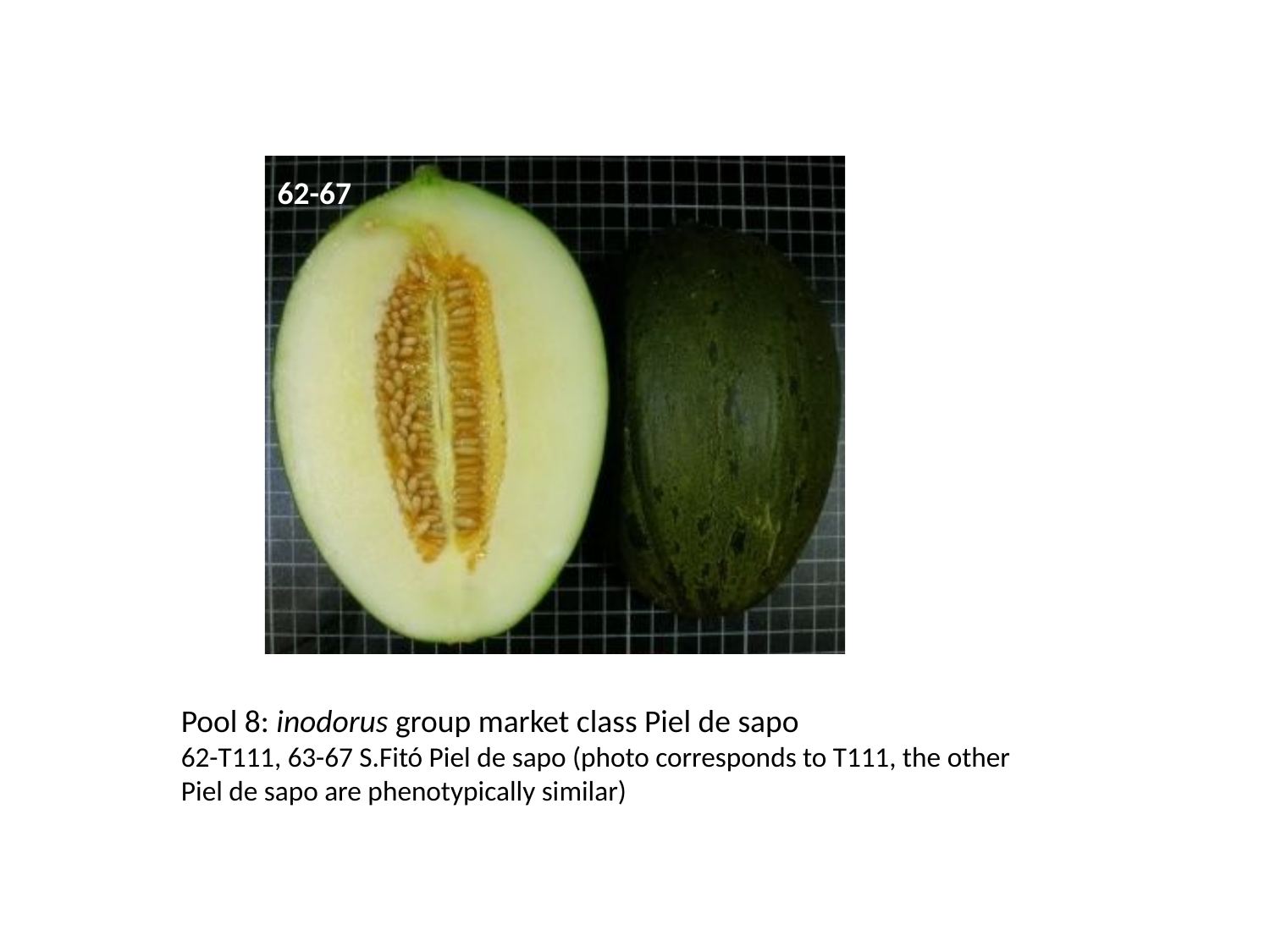

62-67
Pool 8: inodorus group market class Piel de sapo
62-T111, 63-67 S.Fitó Piel de sapo (photo corresponds to T111, the other Piel de sapo are phenotypically similar)
